# Pleuroparenchymal Fibroelastosis With Progressive Bronchiectasis and Apical Pneumothoraces in a Patient With Severe Chronic Obstructive Pulmonary Disease and Chronic Pseudomonas Colonization: A Case Report

**DOI:** 10.7759/cureus.93247

**Published:** 2025-09-26

**Authors:** Shayekh Ferdoush, Ridwan Shahnewaz, Mustain Jawad, Morad Albarouni, Farmina Ahmed

**Affiliations:** 1 Respiratory Medicine, University Hospitals Bristol and Weston NHS Foundation Trust, Weston-super-Mare, GBR; 2 Intensive Care Unit, University Hospitals Bristol and Weston NHS Foundation Trust, Weston-super-Mare, GBR; 3 Internal Medicine, Weston General Hospital, Weston-super-Mare, GBR; 4 Internal Medicine, Bangabandhu Sheikh Mujib Medical University, Dhaka, BGD

**Keywords:** apical fibrosis, bronchiectasis, copd, pleuroparenchymal fibroelastosis, ppfe

## Abstract

Pleuroparenchymal fibroelastosis (PPFE) is a rare condition of progressive interstitial lung disease marked by bilateral upper lobe pneumothoraces and fibrosis. We present the case of a 73-year-old female with a background of bronchiectasis, chronic obstructive pulmonary disease (COPD), and long-standing *Pseudomonas* colonization, who presented with progressive shortness of breath, chest tightness, and cough. Imaging revealed bilateral apical pneumothoraces and progressive bronchiectatic and fibrotic changes. Sputum cultures grew *Escherichia coli*, *Achromobacter* species, and *Candida*. A diagnosis of PPFE was made based on characteristic clinical and radiographic findings. After discussion in the multidisciplinary team (MDT) and confirmation of the diagnosis, given the non-progressive nature of the pneumothoraces and her poor functional reserve, conservative management was advised. This case highlights the complexity of managing advanced respiratory disease in a severely malnourished patient with chronic infection and multiple comorbidities.

## Introduction

Pleuroparenchymal fibroelastosis (PPFE) is a rare and under-recognized subtype of interstitial lung disease (ILD), characterized by dense subpleural fibroelastosis and pleural thickening, predominantly affecting the upper lobes [1,2]. PPFE has, in general, a prevalence of <1% of all ILDs with a slight male predominance. Patients typically present with progressive breathlessness and a dry cough. Diagnosis requires high-resolution CT of the thorax, which reveals characteristic upper lobe pleural thickening and fibrosis and bilateral pneumothoraces. There is no disease-specific treatment, and management is largely supportive, focusing on symptom control. Recurrent respiratory infections and progressive pneumothorax are common complications that significantly impact prognosis.

Bronchiectasis leads to chronic inflammation, microbial colonization, and architectural airway distortion [3]. In chronic cases, particularly involving *Pseudomonas aeruginosa* colonization, repeated inflammation may promote fibrotic remodelling and secondary PPFE.

Radiologically, PPFE is marked by apical pleural thickening, subpleural fibrosis, volume loss, traction bronchiectasis, and pneumothoraces [4]. In contrast to other ILDs, PPFE is notably associated with recurrent or persistent pneumothorax due to stiffened, fragile subpleural tissue [5].

The development of pneumothorax, although uncommon in bronchiectasis, further complicates the clinical course and therapeutic decision-making. This case report presents a patient with advanced bronchiectasis, chronic colonization, and chronic obstructive pulmonary disease (COPD) who developed bilateral apical pneumothoraces and worsening respiratory symptoms, offering insight into the multidisciplinary considerations required in such complex cases.

## Case presentation

A 73-year-old female was admitted with a four-week history of worsening chest tightness, shortness of breath, and productive cough. She reported a significant unintentional weight loss of 18 kg over 16 months. She has a background history of bronchiectasis with *Pseudomonas* colonization (initially identified in 2019), severe COPD (on 3 L/min ambulatory oxygen at home), and osteoporosis. She was an ex-smoker and quit smoking 25 years ago. There was no history of occupational exposure to asbestos or silica.

**Figure 1:**
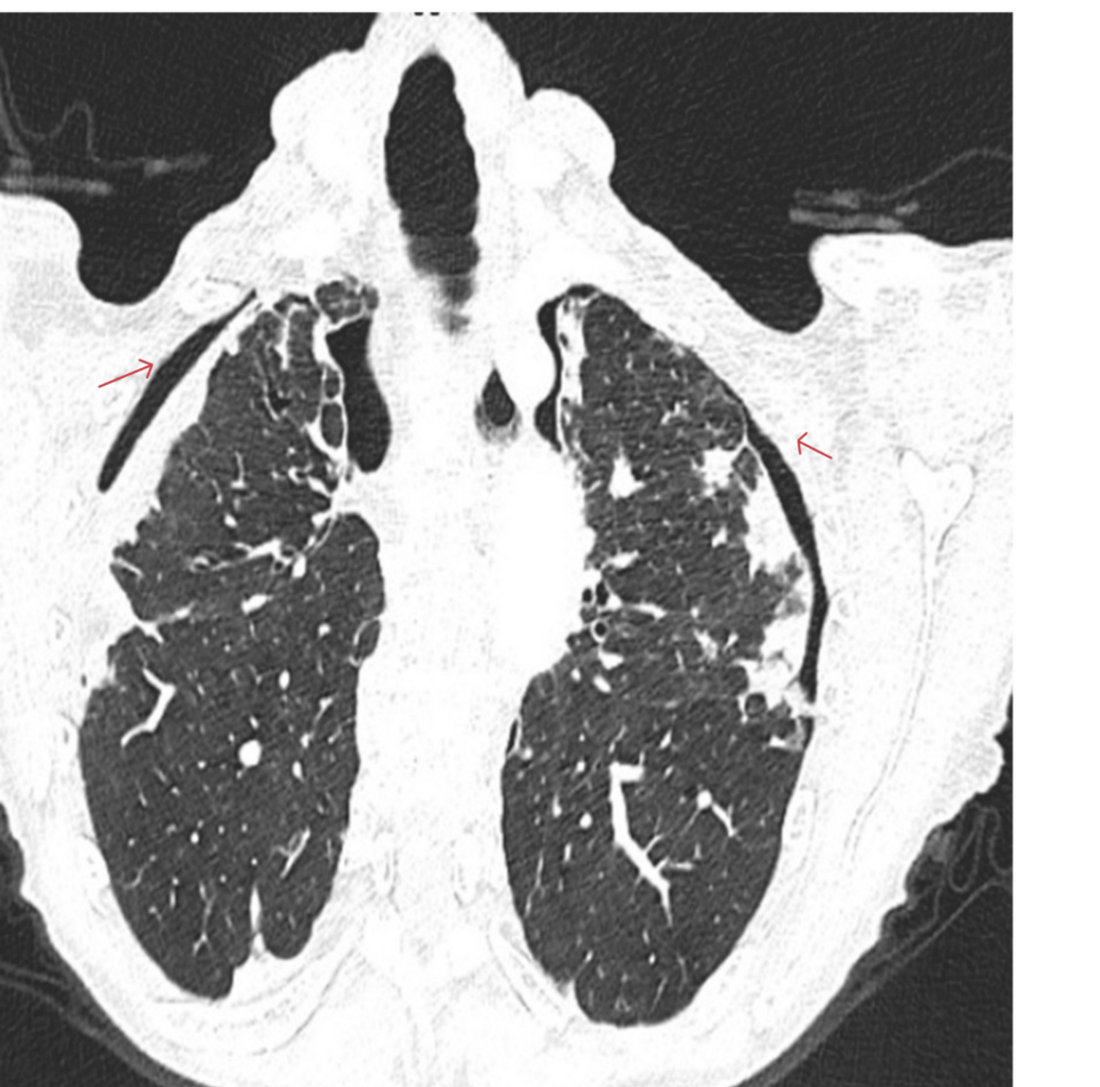
CT of the thorax with contrast showing bilateral (B/L) pneumothoraces (red arrows) with pleural thickening and fibrotic changes.

The patient was reviewed in the community by her general practitioner (GP), who had requested a CT thorax with contrast due to her worsening symptoms and breathlessness. The scan revealed progressive bronchiectasis with fibrotic changes and a rim of air at both lung apices, consistent with bilateral apical pneumothoraces (Figure 1). She was then referred to the respiratory team.

Following several missed outpatient appointments, she was admitted with further clinical deterioration. On examination, she appeared cachectic, with a BMI of 14.16, tachypnoea, and reduced breath sounds bilaterally.

**Figure 2:**
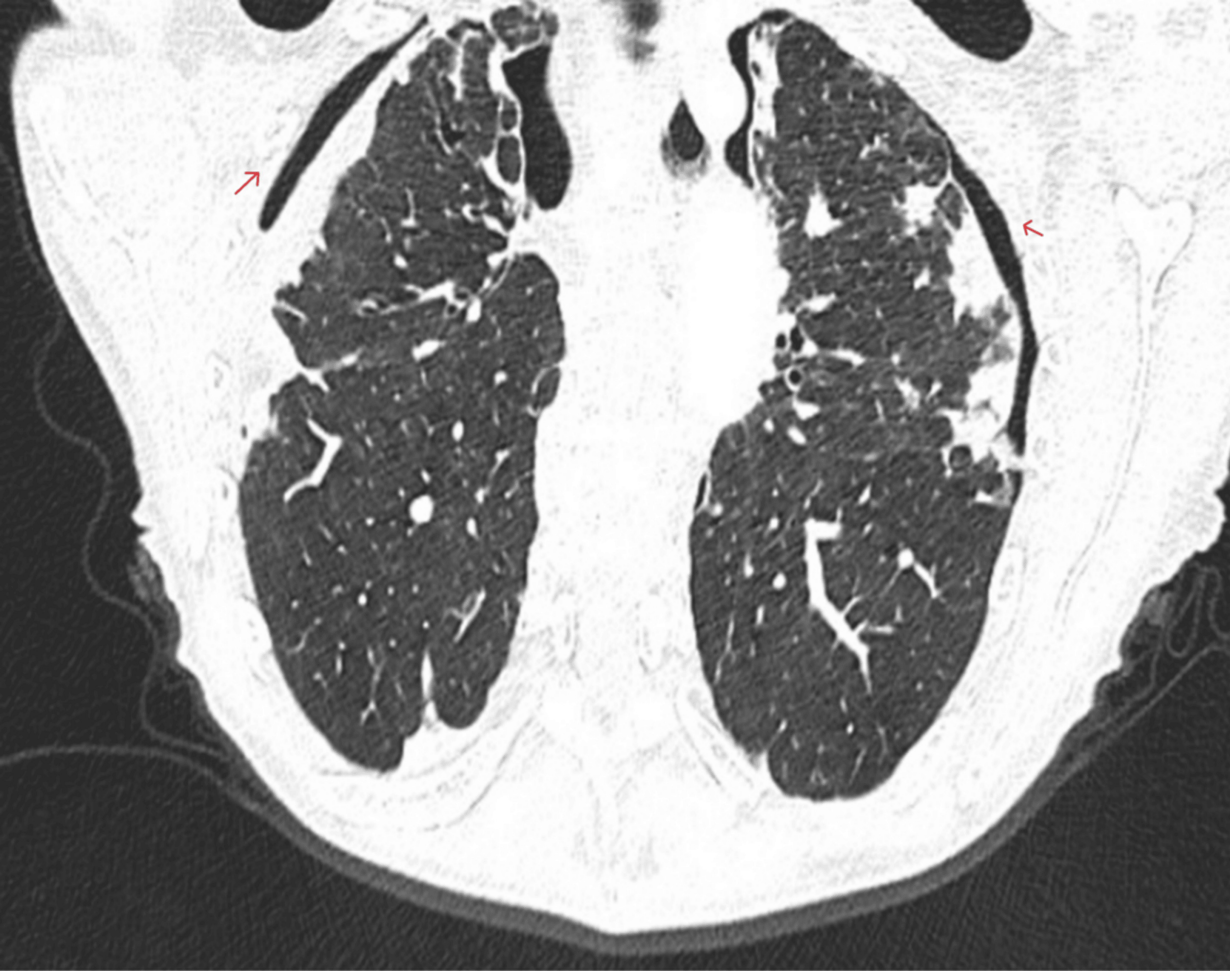
CT pulmonary angiogram showing apical pneumothoraces (red arrows), which are persistent and stable compared to the previous CT thorax with contrast.

Empirical antibiotic therapy with amoxicillin and clavulanic acid was initiated for a presumed infective exacerbation of bronchiectasis. The patient had previously been treated with levofloxacin. Sputum cultures revealed mixed growth of *Escherichia coli*, *Achromobacter xylosoxidans* species, and *Candida* (Table 1). A CT thoracic angiogram was performed on admission (four months after the initial CT thorax with contrast requested by the GP) to investigate chest tightness and pain. This confirmed stable apical pneumothoraces without evidence of pulmonary embolism or other acute vascular pathology (Figure 2). The presence of persistent, stable apical bilateral pneumothoraces also supports the diagnosis of PPFE. All other blood tests, including the autoimmune profile (antinuclear antibody (ANA), antineutrophil cytoplasmic antibody (ANCA), myositis panel, and scleroderma immune blot), were normal, and sputum cultures were negative for *Mycobacterium tuberculosis *(Table 1).

**Table 1:** Investigations of the patient (routine blood tests, autoimmune profile, and sputum testing for MTB) CRP: C-reactive protein; WBC: white blood cells; ANA: antinuclear antibody; ANCA: antineutrophil cytoplasmic antibody; MTB: *Mycobacterium tuberculosis*

Investigations	Findings	Normal Values
Sputum for MTB	No growth	-
CRP	17 mg/dL	<1 mg/dL
WBC	10.9× 10^9^/L	4-11 × 10^9^/L
ANA, ANCA	Negative	-
Myositis and scleroderma immune blot	Negative	-

Thoracic surgeons were consulted for bilateral pneumothoraces, and they recommended conservative management, given the small size and stable appearance of the pneumothoraces and the patient’s poor surgical candidacy. Her medication regimen included formoterol fumarate/beclometasone inhaler, carbocisteine, omeprazole, amitriptyline, salbutamol, codeine phosphate/paracetamol, and azithromycin for prophylaxis.

Her case was subsequently discussed at the weekly ILD multidisciplinary team (MDT) meeting, which confirmed the diagnosis of PPFE. A multidisciplinary approach was adopted, involving respiratory medicine, dietetics, palliative care, and physiotherapy teams. Nutritional support and pulmonary rehabilitation were initiated, and palliative care input was considered due to the high symptom burden and poor prognosis.

## Discussion

This case illustrates diagnostic and therapeutic challenges involving coexisting COPD, chronic bronchiectasis, and PPFE. The radiological pattern of upper lobe fibrosis, pleural thickening, and pneumothorax is characteristic of PPFE [6,7]. PPFE is increasingly reported in association with bronchiectasis, especially when complicated by chronic infection. Repeated episodes of inflammation and colonization with pathogens such as *Pseudomonas*, *Achromobacter*, and *E. coli *may contribute to ongoing alveolar damage and fibrotic remodeling [5,8,9].

Bilateral apical pneumothoraces, uncommon in most ILDs, are a recognized feature of PPFE. They are thought to arise due to increased pleural stiffness, loss of elastic recoil, and parenchymal fragility [4,10]. These pneumothoraces may remain stable and asymptomatic, especially in frail patients with poor reserve.

Our patient’s malnutrition was a significant concern, with a BMI of 14.16. Malnutrition in COPD and ILD is associated with poor immune function, reduced respiratory muscle strength, and higher mortality [10,11]. Nutritional optimization and pulmonary rehabilitation remain crucial supportive interventions.

Given the patient’s severe frailty, poor surgical candidacy, and stable imaging findings, conservative management was deemed most appropriate. Lung transplantation is the only definitive treatment for PPFE, but it is not feasible in most elderly or comorbid patients [12].

This case also underscores the importance of continuity of care. Missed follow-up appointments and delays in intervention may have contributed to progression. Integration of community respiratory services and enhanced patient engagement could improve outcomes in such chronic conditions.

## Conclusions

This case illustrates the complex interplay between PPFE, advanced bronchiectasis, chronic infection, and COPD, culminating in respiratory decline complicated by apical pneumothoraces. A multidisciplinary, patient-centred approach focusing on symptom control, nutritional support, and conservative management is crucial in such cases. Clinicians should remain vigilant for complications in patients with structural lung disease and ensure timely intervention to prevent further deterioration.
